# A Rare Finding of Falciform Ligament Thrombosis as a Sequel of Acute Pancreatitis

**DOI:** 10.1155/2017/2879568

**Published:** 2017-12-18

**Authors:** H. Q. C. Lim, X. W. J. Lee, N. Mathias

**Affiliations:** Hairmyres Hospital, Glasgow G75 8RG, UK

## Abstract

Falciform ligament (remnant of umbilical vein) is an anatomical structure that connects the liver to the anterior abdominal wall. This case reports a rare clinical presentation of falciform ligament thrombosis as a consequence of acute gallstone pancreatitis, in a patient with noncirrhotic liver. A 55-year-old female with a history of cholelithiasis was admitted with abdominal pain. Biochemistry profile showed hyperamylasemia and deranged liver function tests. Computerized Tomography (CT) revealed a 3 cm attenuated structure that can be traced up to the left portal vein, which represents an acute thrombosis of the falciform ligament. The patient was treated with Tinzaparin and subsequently anticoagulated. She subsequently had a laparoscopic cholecystectomy and made an uneventful recovery. We suspect that pancreatitis caused thrombophlebitis subsequently leading to recanalization and thrombosis of the umbilical vein. Falciform ligament thrombosis is a rare and poorly described complication following pancreatitis which clinicians and radiologists should be aware of.

## 1. Introduction

The falciform ligament of the liver is a fibrous remnant of the obliterated umbilical vein [1, 2]. The umbilical vein and artery play a vital role during fetal growth. These structures allow the exchange of materials between fetal and maternal circulation. The umbilical vein divides into two structures at the liver (ductus venosus and a branch that communicates with the hepatic portal vein). During fetal development, the majority of oxygenated blood travels via the umbilical vein into the ductus venosus that subsequently drains into the inferior vena cava [2]. When renal, pulmonary, and digestive functions begin to function at birth, the umbilical vein collapses and remains as the falciform ligament.

Pancreatitis can potentially cause both systemic and local complications. Diseases involving the falciform ligament are extremely rare and recanalization of the umbilical vein has only been described in patients with portal hypertension [3]. We describe an unusual case report of isolated falciform ligament thrombosis as a sequel of acute pancreatitis.

## 2. Case Presentation

A 55-year-old female presented with a one-day history of colicky pain in the epigastric and right upper quadrant area with associated vomiting. The pain did not resolve with regular cocodamol 8/500 mg (2 tablets). She has a background of cholelithiasis and her symptoms were described as similar. She has no history of pancreatitis. She is a nonsmoker and consumes nine units of alcohol per week. There is no family history of bleeding or clotting disorders. Her only regular medication is hormone replacement therapy* (estradiol, norethisterone)*.

On admission, her basic observations were normal: temperature (36.8°C), blood pressure (117/79 mmHg), heart rate (73 beats per minute), and alert and oriented. Abdominal examination revealed point tenderness in the right upper quadrant, no organomegaly, or peritonism.

Admission laboratory investigations revealed deranged liver function tests: alanine aminotransferase (ALT) (275 U/L), bilirubin (54 umol/L), hyperamylasemia (1900 U/L), normal alkaline phosphatase (80 U/L), normal C-reactive protein (<6 mg/L), and raised white cell count (18 × 10^9^/L). Abdominal ultrasound revealed cholelithiasis with no evidence of common bile duct obstruction. The patient was subsequently treated for gallstone pancreatitis with intravenous fluid therapy and analgesia.

**Figure 1:**
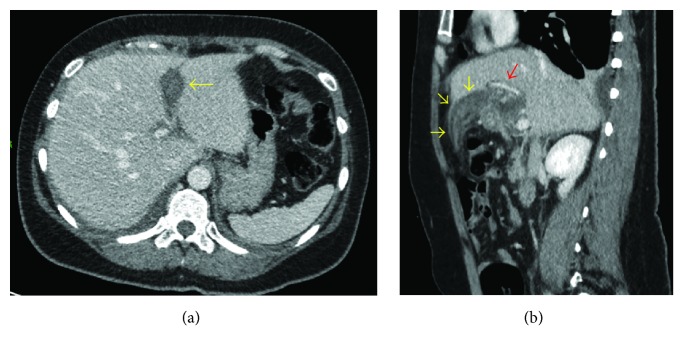
Abdominal axial (a) and sagittal (b) CT scan showing thrombosed falciform ligament (yellow arrows) with patent left portal vein (red arrow).

**Figure 2:**
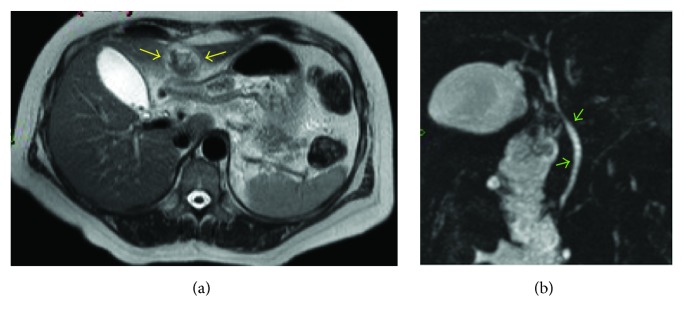
MRCP (magnetic resonance cholangiopancreatography). Axial view (a) showing thrombosed falciform ligament (yellow arrows). Projective coronal view (b) showing patent bile duct with no stones (green arrows).

Three days after admission, she had episodes of pyrexia (temperature 38.2°C) with elevated inflammatory markers (C-reactive protein 255 mg/L and white cell count 19 × 10^9^/L). She was started on intravenous antibiotics empirically (Amoxicillin 1 g three times a times a day, Metronidazole 500 mg three times a day, and Gentamicin as per weight and renal function) to treat possible biliary sepsis and/or necrotizing pancreatitis. Liver function tests had improved with bilirubin 20 umol/L, ALT 81 U/L, and amylase of 143 U/L. Computerized Tomography (CT) scan of her abdomen and pelvis with contrast (Figure 1) was performed to confirm our diagnosis but instead it revealed a thin-walled gall bladder, stranding adjacent to the pancreatic tail and a 3 cm reduced attenuation structure, which was thought to represent an acute thrombosis of the umbilical vein. There was no evidence of collection. Magnetic resonance cholangiopancreatography (MRCP) (Figure 2) was subsequently performed which confirmed the above findings and no biliary duct stones were identified. Intravenous antibiotics were immediately discontinued as per protocol as no source of sepsis was found from imaging.

Patient coagulation parameters (prothrombin time, partial thrombin time, International Normalized Ratio, platelets, and fibrinogen) were normal since admission. A thrombophilia screen was subsequently carried out and was normal. She was treated with Tinzaparin 12000 units (treatment dose) and was discharged home after a week with warfarin (target INR of 2.0–3.0) for a target period of 6 months and hormone replacement therapy discontinued.

**Figure 3:**
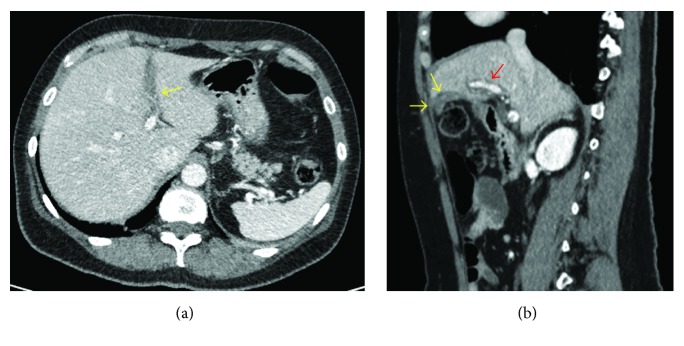
Abdominal axial (a) and sagittal (b) CT scan showing resolution of falciform ligament thrombosis (yellow arrows) with patent left portal vein (red arrow).

**Figure 4:**
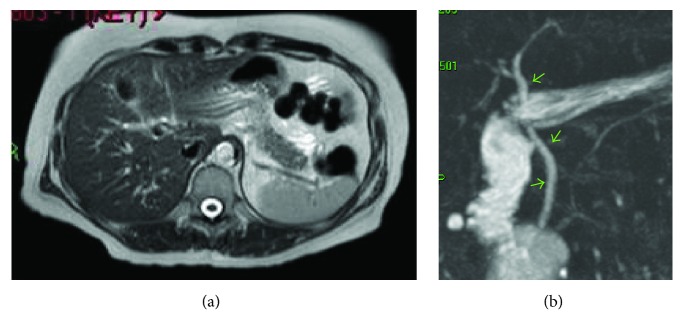
MRCP axial view (a) showing postcholecystectomy and resolved falciform ligament thrombosis. Projective coronal view (b) showing patent bile duct (green arrows).

Four months after her hospital admission, she had a second episode of pancreatitis (hyperamylasemia of 1819 U/L). CT scan of the abdomen and pelvis with contrast (Figure 3) demonstrated ongoing cholecystitis with a resolving falciform ligament thrombosis. Laparoscopic cholecystectomy was carried out uneventfully and operative cholangiogram revealed no stones in the common bile duct (CBD). Histology of the gall bladder revealed features of chronic cholecystitis with multiple yellow mulberry stones ranging from 3 to 8 mm in diameter. A MRCP scan (Figure 4) was carried out 8 weeks after procedure and revealed no biliary duct stones or strictures and a complete resolution of falciform ligament thrombosis.

## 3. Discussion

Isolated umbilical vein thrombosis in the adult population is a rare occurrence. To the best of our knowledge, there have been no reported cases in the literature. Incidental isolated umbilical vein thrombosis in neonates has occurred as a consequence following umbilical vein catheterisation [4, 5]. Pancreatitis is an inflammatory process and is well documented to potentially cause venous and arterial vascular complications. These complications are also noted to be more common in alcohol induced, necrotizing, and chronic pancreatitis [6]. Studies have stated that venous thrombosis following pancreatitis is frequent and typically manifests as splanchnic vein thrombosis (SVT), predominantly involving splenic vein (SpIV) [7]. This thrombosis frequently resolves spontaneously after the resolution of inflammatory disease. Clinically, SVT in pancreatitis is becoming more common with advancement of imaging technology. One recent meta-analysis demonstrated that 13.6% of pancreatitis had SVT, 6.2% had portal vein thrombosis (PVT), 11.2% had SplV thrombosis (SplVT), and 2.7% had mesenteric vein thrombosis (MVT). It also identified that the prevalence of SVT in pancreatitis in Europe reached 16.9%, which is the highest among the three continents compared (Europe, America, and Asia) [8].

The causes of PVT can be primarily classified into either local causes or systemic prothrombotic states. Various reports [9–11] described PVT following pancreatitis; however acute recanalization of the paraumbilical vein is rare. The umbilical vein however has been speculated to spontaneously recanalize during portal hypertension to serve as a hepatofugal, decompressing collateral [12]. The peculiarity of this case presentation is based on the fact there has been acute thrombosis of the falciform ligament in a noncirrhotic liver with a patent portal venous system.

We suspect that this patient may have thrombophlebitis secondary to pancreatitis rather than an infective source. This has been supported by radiological and biochemical findings (CT describing no evidence of cholecystitis and improving liver function tests). The paraumbilical vein originates from the umbilical portion of the left portal vein in the falciform ligament and proceeds to the umbilicus and periumbilical veins [13]. We hypothesise that the effects of pancreatitis in this patient was thrombophlebitis which may have spread via the portal venous system into the falciform ligament subsequently leading to spontaneous recanalization and acute thrombosis.

At present, review of the previous studies and case reports outlined that both CT and MRI can be used for diagnosis. However, its appearance on both imaging modalities varies with the thrombus age. Acute clot appears hypodense or isodense to adjacent liver structures on CT while showing as high intensity signals on both T1 and T2 weighted MRI sequences. MRI has been noted to be the most sensitive imaging modality for the detection of PVT but its appearance is once again dependant on thrombus age and specific MRI sequence. As time progresses, there is a loss of T2 weighted signal on MRI [9, 14].

We extrapolated treatment recommendations from cases [15–18] of PVT following pancreatitis and treated her with early anticoagulation therapy. There has been no evidence to suggest that initial thrombolysis should be given in preference to anticoagulation for patient with PVT and anticoagulation did not increase the risk of bleeding. However, there have been reported cases where thrombolysis has been shown to be effective only when initial anticoagulation therapy has failed [10]. We agree with other studies [10, 11] and recommend that anticoagulant therapy should be given for only six months and continued only if an underlying thrombophilia has been identified in umbilical vein thrombosis.

## 4. Conclusion

In conclusion, falciform ligament thrombosis is a rare and poorly differentiated complication following pancreatitis which both clinicians and radiologists should be aware of. In this setting, it is important to exclude falciform ligament thrombosis as a differential diagnosis in a patient with ongoing abdominal pain despite adequate management of the pancreatitis. The acute diagnosis can be well demonstrated on both contrast CT and MRI, and we recommend early anticoagulation to allow recanalization.
